# Excess mortality is not self-interpreting: Baseline choice as a governance issue in public health analytics

**DOI:** 10.1177/14034948261424605

**Published:** 2026-03-01

**Authors:** Jörn Klein

**Affiliations:** Department of Nursing and Health Sciences, University of South-Eastern Norway, Porsgrunn, Norway

**Keywords:** Excess mortality, all-cause mortality surveillance, baseline selection, public health analytics, risk communication

## Abstract

Interpretation of excess mortality depends critically on baseline choice. This commentary examines the recent debate on all-cause mortality in Norway in 2024 and argues that it reflects a conflation of two distinct purposes: real-time surveillance using updated baselines and assessment of whether mortality has returned to the pre-pandemic improvement trajectory. These correspond to different estimands and are not interchangeable. Baseline redefinition has predictable inferential and communicative consequences, particularly in public-facing outputs. I argue that the main risk lies not in modeling choices themselves, but in insufficiently explicit communication. Transparent reporting with clearly labeled baselines is essential to avoid interpretive drift.

All-cause mortality surveillance has become a central instrument for situational awareness during and after the COVID-19 pandemic. The debate between White et al. and Knudsen et al. is therefore not a narrow technical dispute; it concerns a recurring governance problem in public health analytics: the same indicator (“excess mortality”) is routinely asked to serve two distinct purposes, and when the analytic baseline is changed without explicit framing, public interpretation can drift from “routine surveillance” into “policy evaluation” or, conversely, into unwarranted reassurance [1,2].

## Two legitimate questions, two different estimands

The disagreement is partly about the target quantity being estimated (an estimand): (i) a real-time surveillance contrast, where 2024 mortality is assessed against an expected value derived from a recently updated reference period; versus (ii) a counterfactual trend-break contrast, where 2024 mortality is assessed against what would be expected if the pre-2020 mortality improvement trajectory had continued. These are both legitimate, but they are not interchangeable.

Knudsen et al. defend the Norwegian Institute of Public Health (NIPH) approach as a real-time surveillance model intended to “monitor and describe mortality trends in real time – not to evaluate health measures or pandemic strategies” [1]. Their argument is coherent insofar as operational surveillance requires timely expected values and uncertainty bands, and it is reasonable for surveillance systems to evolve as COVID-19 becomes one of several endemic drivers of mortality variability.

White et al., however, are not primarily disputing whether NIPH’s approach can support real-time monitoring. Their core claim is that the updated baseline used for 2024 is not suitable for assessing whether the long-standing pre-pandemic decline in mortality has resumed [2]. In other words, White et al. are targeting a different estimand: detection of a trend break/level shift relative to the pre-2020 trajectory. Both questions are legitimate, but they are not interchangeable—and answers to one should not be presented as if they answer the other.

## Baseline redefinition is not a neutral “technical update”

The most important methodological point in this exchange is straightforward: baseline choice is a modeling decision with predictable inferential consequences. If a post-pandemic year with substantial excess mortality is incorporated into the reference period for expected mortality, the counterfactual for a subsequent year will, by construction, tend to move upward (and uncertainty may widen), making “within expected levels” more likely.

Knudsen et al. explicitly criticize the “conservative approach” of White et al. because it carries forward a predicted 2023 value rather than using observed 2023 mortality to inform expected 2024 mortality, implying an underlying baseline effectively ending in 2019 [1]. Knudsen et al. are correct that this embeds a strong assumption: that 2020–2024 mortality can be predicted from 2010–2019 patterns and that pandemic-era deviations are principally pandemic-related.

However, that is precisely why this is not a matter that can be resolved by appealing to the “purpose” of surveillance alone. If an agency changes baselines, then—particularly in public-facing outputs—the agency has changed the meaning of the headline claim, unless it clearly distinguishes (a) routine monitoring relative to a recently updated expectation, from (b) assessment of whether mortality has returned to the pre-pandemic improvement path.

## Communication is the practical locus of harm

White et al. argue that NIPH combined statements generated under different baselines across years and that this can create a misleading narrative (“mortality back to pre-pandemic levels”), because the reader is not shown that a key parameter—the baseline—has shifted [2]. This is an argument about interpretability and public inference, not merely about statistical technique.

Knudsen et al. respond that NIPH does not evaluate pandemic strategy [1]. Yet White et al. make a credible communication critique: when a press release explicitly contrasts “periods with high mortality during the COVID-19 pandemic” with “within expected levels” in 2024, many readers will interpret this as a comparative judgement that implicitly bears on policy success, regardless of the stated intention [2].

This is the central governance tension: public health agencies cannot assume that technical intent will control public interpretation. If outputs are framed in comparative language across periods with different strategies, then the communication functions as evaluation. If, conversely, the purpose is strictly surveillance, then the communication should be constrained to that purpose and should avoid cross-period narrative claims that depend on non-comparable baselines.

## What would constitute a constructive resolution?

A technically and institutionally robust resolution does not require one side to “win.” It requires transparent pluralism in reporting:

1. *Parallel reporting with multiple baselines*. Agencies can—and should—publish side-by-side estimates for (i) a surveillance baseline updated to recent years, and (ii) a pre-pandemic counterfactual baseline designed to test for trend breaks [2]. This directly addresses White and colleagues’ call for sensitivity analyses while preserving Knudsen and colleagues’ legitimate surveillance aim [1].
2. *Explicit labeling of estimand and baseline in every headline output*. Public-facing statements should specify whether the claim is “within expected levels relative to an updated surveillance baseline” or “consistent with return to the pre-pandemic improvement trajectory” (if such an analysis is actually performed). Without this, a baseline switch becomes a silent shift in meaning.
3. *A minimum communication standard for baseline changes*. Baseline revisions should trigger a short, mandatory “model change notice” in public materials: what changed, why it changed, how it changes interpretation, and what trends remain unknown.
4. *Decomposition beyond all-cause totals*. Both parties are correct that all-cause mortality is a blunt instrument for causal claims [1,2]. But this also means that reassurance narratives should be cautious. Trend breaks—if present—require cause-of-death decomposition, age-standardized reporting, and examination of competing explanations (healthcare access, influenza seasons, long-term sequelae, demographic shifts, coding practice, and other contributors). As White et al. emphasize, custodians of cause-of-death registries are uniquely positioned to lead this work.

## Conclusion

The exchange between White et al. and Knudsen et al. surfaces a broader lesson for Scandinavian public health practice: excess mortality metrics are not self-interpreting. Baselines encode the question being asked. Surveillance baselines can be appropriate for operational monitoring; counterfactual baselines are required to assess whether pre-pandemic improvement has resumed. The critical requirement is that agencies do not mix conclusions across these estimands without transparent labeling. The remedy is not more rhetoric about intent, but routine sensitivity reporting and disciplined public communication [1,2].
